# P-1789. Implementation of Pathogen Independent Model Informed Precision AUC-based Dosing of Vancomycin at University Hospital Wuerzburg (UKW)

**DOI:** 10.1093/ofid/ofae631.1952

**Published:** 2025-01-29

**Authors:** Laura C Gundlach, Güzin Surat, Mareike Kunkel, Oliver Scherf-Clavel

**Affiliations:** University Hospital Wuerzburg, Wuerzburg, Bayern, Germany; University Hospital Würzburg, Würzburg, Bayern, Germany; University Hospital Wuerzburg, Wuerzburg, Bayern, Germany; Faculty for Chemistry and Pharmacy, Ludwid-Maximilians-University Munich, Munich, Bayern, Germany

## Abstract

**Background:**

The IDSA guideline concerning monitoring vancomycin in MRSA infections, published in March 2020, recommends model informed precision AUC based dosing for vancomycin. The application of pharmacometrics software with Bayesian calculating is recommended. The summary of product characteristics of vancomycin, issued by EMA (European Medicines Agency), includes also details regarding model informed precision dosing for vancomycin therapies, irrespective of targeted or identified pathogens. Due to the low prevalence of MRSA infections at UKW, model informed precision AUC-based dosing has been instituted for all parenteral vancomycin therapies.

**Methods:**

The target for vancomycin AUC/MIC (minimum inhibitory concentration) is a range of 400 – 600 mg*h/L over 24 hours. Individual patient covariates, personal dosing information and vancomycin concentrations were converted into dosing recommendations with InsightRX. In order to record the effect of the intervention, the target attainment and the occurrence of AKI (acute kidney injury, defined as an increase in serum creatinine of ≥ 0.3 mg/dL within 48 h) is recorded 18 months before and 12 months after the implementation of the new dosing strategy.

**Results:**

Since October 2022, a workflow for model informed AUC-based dosing has been launched across 12 wards - mainly intensive care units - encompassing 215 beds. In the conventional dosing strategy according to trough dosing, AKI occurred in 60 of 216 therapies, while AKI was detected in 8 out of 57 AUC-guided vancomycin therapies resulting in an odds ratio of 2.32 [CI 95 % 1.08 – 5.59]. Significantly more AUC guided therapies were in the therapeutic range of 400 – 600 mg*h/L after 120 hours than trough-based vancomycin therapies reached the target [55.1 % vs 70.2 %, OR 0.52, CI 95 % 0.27 – 0.97]. Additionally, more trough-based therapies were in a supratherapeutic range than AUC guided therapies [24.5 % vs 8.8 %, OR 3.23, CI 95 % 1.35 – 9.98]

**Conclusion:**

The unique concept of pathogen independent model-informed AUC-based dosing of vancomycin was successfully integrated into the clinical routine work. Early dose individualization due to AUC-based dosing can contribute to increase drug therapy safety and improved patient safety for vancomycin therapies.

**Disclosures:**

**All Authors**: No reported disclosures

